# Draft genome of the *Klebsiella pneumoniae* 24Kpn33 and complete sequence of its pCOL-1, a plasmid related to the *bla*_KPC-2_ acquisition in *Pseudomonas aeruginosa*

**DOI:** 10.1128/mra.00071-24

**Published:** 2024-03-18

**Authors:** Deisy Abril, Duway Nicolas Lesmes-Leon, Ricaurte Alejandro Marquez-Ortiz, Aura Lucía Leal, Catalina Tovar-Acero, Zayda Lorena Corredor Rozo, Natasha Vanegas Gómez, Javier Escobar-Perez

**Affiliations:** 1 Bacterial Molecular Genetics Laboratory - LGMB, Universidad El Bosque, Bogotá, Colombia; 2 University of Kaiserslautern-Landau (RPTU), Kaiserslautern, Germany; 3 German Research Center for Artificial Intelligence (DFKI), Kaiserslautern, Germany; 4 Facultad de Medicina y Grupo de Investigación en Enfermedades Infecciosas, Universidad Nacional de Colombia, Bogotá, Colombia; 5 Departamento de Patología y Laboratorios, Fundación Santa Fe de Bogotá, Bogotá, Colombia; 6 Grupo de Investigación en Enfermedades Tropicales y Resistencia Bacteriana, Universidad del Sinú, Montería, Colombia; 7 The i3 Institute, Faculty of Science, University of Technology, Sydney, Australia; Rochester Institute of Technology, Rochester, New York, USA

**Keywords:** *bla*_KPC-2_, pCOL-1, Tn*4401b*, interspecies dissemination

## Abstract

We report the draft genome of a clinical multi-resistant *Klebsiella pneumoniae* (24Kpn33) isolate, whose genome (5.7 Mbp) harbored 17 antibiotic resistance genes, including *bla*_KPC-2_. Notably, this gene was mobilized within the IncP-6 pCOL-1 plasmid, the first genetic platform related to the acquisition and dissemination of the *bla*_KPC-2_ in *Pseudomonas aeruginosa*.

## ANNOUNCEMENT

*Klebsiella pneumoniae* carbapenemase (KPC) is one of the most worldwide spread carbapenemases in gram-negative bacteria. It was described for the first time in *K. pneumoniae* in 1996 and rapidly dispersed to many countries (1, 2). The first detection of KPC in *Pseudomonas aeruginosa* was carried out in Colombia (2006) due to a suggested horizontal transfer of a 32-kb-size IncP-6-plasmid, pCOL-1, harboring *bla*_KPC_ into a Tn*4401b* (3, 4). Since then, several worldwide studies of *bla*_KPC_*-*harboring *P. aeruginosa* (KPC*-Pa*) isolates had been related with Tn*4401* variants and recently with novel mobilization platforms like NTE_KPC_ (non-Tn*4401* elements)—https://maphub.net/LGMB/KPC-Pseudomonas-aeruginosa-LGMB— both genetic elements detected in plasmids and in chromosome (5). Despite KPC connection with *K. pneumoniae,* pCOL-1 had never been found in a *K. pneumoniae* or Enterobacterales isolates as evidence of a possible *bla*_KPC_ mobilization to *P. aeruginosa*.

**Fig 1:**
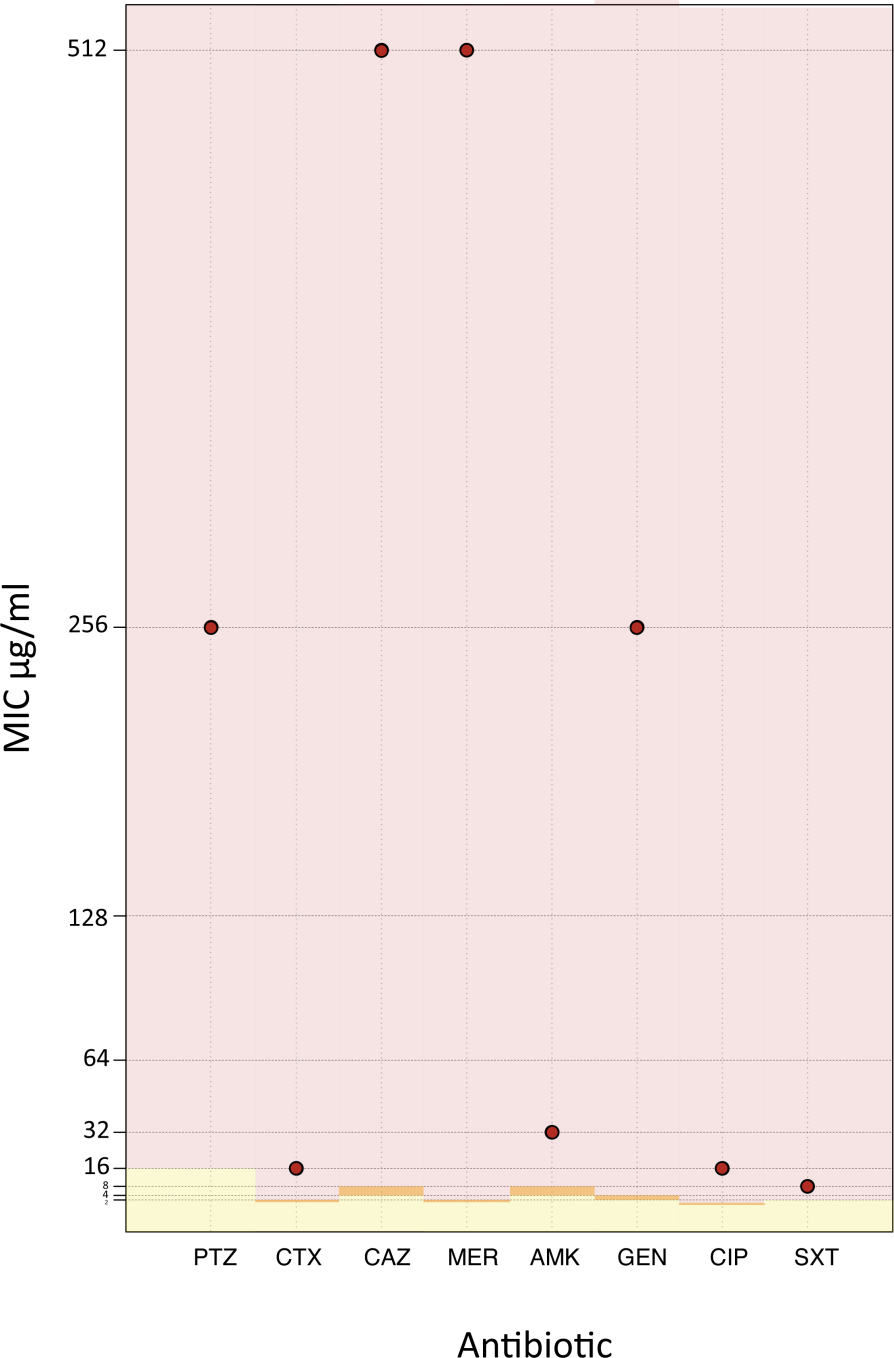
Minimal inhibitory concentration of clinical isolate 24Kpn33 for principal antibiotic families. MIC breakpoints of susceptible (yellow), intermediate (orange), and resistant (red) were illustrated. Red circles indicate the MIC for β-lactams [piperacillin-tazobactam (PTZ), cefotaxime (CTX), ceftazidime (CAZ), meropenem (MER)], aminoglycosides [amikacin (AMK), gentamicin (GEN)], quinolones [ciprofloxacin (CIP)], and sulfonamides [trimethoprim-sulfamethoxazole (SXT)].

In a retrospective whole-genome study, we found the *K. pneumoniae* isolate 24Kpn33, which was recovered from a female patient with pneumonia, who was attended at the Intensive Care Unit of a hospital in Monteria (Colombia) in 2014. With respect to minimal inhibitory concentration (MIC) (using broth microdilution method), 24Kpn33 was resistant to β-lactams (piperacillin-tazobactam: >256 µg/mL, cefotaxime: >16 µg/mL, ceftazidime: 512 µg/mL, meropenem: 512 µg/mL), aminoglycosides (amikacin: 32 µg/mL, gentamicin: 256 µg/mL), quinolones (ciprofloxacin: 16 µg/mL), and sulfonamides (trimethoprim-sulfamethoxazole: 8 µg/mL) (Fig. 1).

For whole-genome sequencing, a paired-end library (350 bp) was created with TruSeq DNA-PCR-free Sample-Preparation kit and sequenced in a MiSeq platform (Illumina). A total of 2,748,118 reads were checked for quality using FastQC v0.11.5 (6) and trimmed with Trimmomatic v0.36 (7). The 2,746,156 filtered reads (>Q20) were assembled with SPAdes v3.10.1 (8). The plasmid pCOL-1 gaps were filled using PCR and Sanger sequencing. *K. pneumoniae* species was confirmed by Kleborate v2.2.0 (9), and multilocus sequence typing (10) was performed on BIGSdb (Institut Pasteur) available on https://bigsdb.pasteur.fr/klebsiella/. Resistance genes were identified by ResFinder (11) with a 90% of threshold and minimum length of 60%; contigs of plasmids were detected by PlasmidFinder (12) and Basic Local Alignment Tool (BLASTn) (13) with Nucleotide NCBI database.

Multiresistant 24Kpn33 (ST 15) isolate has a genome length of 5.7 Mbp distributed in 161 contigs with a GC% of 57.08 (N50 = 208.956/L50 = 10). A total of 5,512 coding DNA sequences, 22 rRNAs, and 85 tRNA were annotated. Regarding to antibiotics resistance, the isolate harbors 17 resistance genes, six to β-lactams: *bla*_SHV-28_, *bla*_TEM-1_, *bla*_CTX-M-15_, *bla*_OXA-1_, *bla*_OXA-9_, and *bla*_KPC-2;_ four to aminoglycosides: *aac(3)IId*, *aadA1*, *aac(6′)-Ib*, and *aac(6′)-II*; and one to fluoroquinolones, trimethophrim, sulfonamides, phenicol, and fosfomycin: *qnrB19, dfrB3, sul1, catB3, fosA*, respectively; and *oqxAB* genes for an efflux pump, in which overexpression promotes resistance to quinoxalines, quinolones, tigecycline, nitrofurantoin, chloramphenicol, detergents, and disinfectants (14).

Regarding to *bla*_KPC-2_ genetic environment, this was associated to a Tn*4401b* in an IncP-6 plasmid with a 99.9% identity with the pCOL-1 from *P. aeruginosa*. This is the first report of a pCOL-1 plasmid in other species. Our finding supports the possible *Klebsiella*-to-*Pseudomonas* inter-species dissemination event of this widely propagated mechanism of carbapenems resistance between these two important clinical pathogens.

## Data Availability

The Sequence Read Archives (SRA) are available in PRJNA1026593, and complete sequence of the genome is available in the DDBJ/EMBL/GenBank public databases under accession number JAYEAB010000035.1.
